# Integrated metabolome-transcriptome analyses reveal key pathways regulating staminate catkin development and pollen maturation in *Betula platyphylla*

**DOI:** 10.3389/fpls.2025.1581560

**Published:** 2025-05-21

**Authors:** Xinying Liu, Jiayuan Shi, Jingyun Zhang, Bello Hassan Jakada, HuiLing Guo, Kehao Zeng, Xingguo Lan

**Affiliations:** Key Laboratory of Saline-Alkali Vegetation Ecology Restoration, Ministry of Education, College of Life Sciences, Northeast Forestry University, Harbin, China

**Keywords:** *Betula platyphylla*, flavonoids, metabolomics, transcriptomics, staminate catkins, pollen maturation

## Abstract

**Introduction:**

Flavonoids are the major metabolites in *Betula platyphylla*. However, our understanding of the role that flavonoids play in staminate catkin development and pollen maturation is still limited.

**Methods and results:**

Here, we performed a metabolome analysis during staminate catkin development and pollen maturation in *B. platyphylla*. These results indicated that mature pollen exhibited significantly high concentrations and diverse profiles of flavonoids. Moreover, using the integrated transcriptomic and metabolomic analyses, we found major metabolites and key genes involved in staminate catkin development and pollen maturation in the flavonoid pathway. Furthermore, WGCNA analysis identified key transcription factors (*BpMYB18*, *BpMYB72*, *BpMYB95*, and *BpMYB109*) as potential regulators in the flavonoid pathway.

**Discussion:**

These findings shed light into the metabolic pathways regulating the development of staminate catkins and pollen maturation in *B. platyphylla*.

## 1 Introduction

*Betula platyphylla* is a monoecious tree species, which produces staminate and pistillate flowers on separate inflorescences (catkins). The staminate and pistillate catkins exhibit heterochronic development. Staminate catkins initiate differentiation in the last summer, forming bright green terminal buds on branches. Following winter dormancy, these catkins transition to dark brown during cold acclimation. Four months later, the staminate catkin began to expand, and after one month the mature pollen is released (Zhang et al., 2023a). The development of the anther is crucial for successful plant reproduction (Wang et al., 2019a). Consequently, the staminate catkin plays a pivotal function in the reproductive success and evolutionary trajectory of *B. platyphylla*. However, the key metabolic pathway in the development of staminate catkin and pollen maturation in *B. platyphylla* remains underexplored. The accumulation of metabolites modulates growth, development, and environmental cues response in plants (Owusu Adjei et al., 2021; Zhang et al., 2024b). In plants, metabolites were divided into primary and secondary metabolites (Hanada et al., 2011). Many compounds are categorized as secondary metabolites, including flavonoids, terpenoids, alkaloids, phenolics, glycosides, tannins, and saponins participating in response to multiple stimuli (Shim et al., 2017; Cao et al., 2021a) and plant organ development (Barnabás et al., 2008; Prasad et al., 2015).

Metabolomic analysis can systematically identify the metabolite changes under varying environmental conditions or different developmental stages of plant organs. Additionally, this approach elucidates the intricate interplay between phenotype, metabolic networks, transcriptomic regulation, function, and plant growth (Duan et al., 2022; Dong and Lin, 2021; Khallouki et al., 2024). While the integration of metabolomic and transcriptomic data can shed light on gene-metabolite interactions, a focus on flavonoids within this framework provides more precise, reliable, and insightful information regarding plant growth and development.

Flavonoids form a large group of secondary metabolites and consist of ring A, ring B, and ring C as a core of the aromatic structure (Tohge et al., 2017). Based on C-ring variation, they are classified as chalcones, flavans, flavones, flavonols, proanthocyanidins, and anthocyanins synthesized through the flavonoid biosynthesis pathway (Winkel-Shirley, 2001). The flavonoid sequestration in plants is conserved (Lepiniec et al., 2006). In plants, the flavonoid biosynthesis pathway features eight branches, however, all eight branches converge on four intermediate metabolites (flavanone, chalcone, leucoanthocyanidin, and dihydroflavonol) (Liu et al., 2021). The phenylalanine ammonialyase (PAL) is the initial enzyme in flavonoid biosynthesis, which plays a major role when plants transition from primary to secondary metabolism (Barros and Dixon, 2020). This enzyme catalyzes L-phenylalanine to cinnamic acid in the first step of the pathway (Kim et al., 2021). Cinnamic acid 4-hydroxylase (C4H) hydroxylates the C4 position of cinnamic acid to produce p-coumaric acid and 4-coumarate, and CoA ligase (4CL) catalyzes cinnamic acid by a condensation reaction to synthesize p-coumaroyl CoA (Santín and Moncalián, 2018). PAL, C4H, and 4CL are often coordinately expressed (Mizutani et al., 1997). Many enzymes participated in the core of the flavonoid pathway, such as chalcone synthase (CHS) which catalyzes p-coumaroyl CoA and malonyl CoA to produce naringenin chalcone (Yonekura-Sakakibara et al., 2019). Chalcone isomerase (CHI) has the catalytic activity to produce naringenin, a key intermediate in the flavonoid biosynthetic pathway (Tohge et al., 2017). Flavanone-3-hydroxylase (F3H), and flavonol 3’-hydroxylase (F3’H) are classified as early biosynthesis genes, playing crucial roles in the initial steps of flavonoid synthesis. The other group, referred to as the late biosynthesis genes, includes dihydroflavonol 4-reductase (DFR), anthocyanidin synthase (ANS), anthocyanidin reductase (ANR), flavonol synthase (FLS) and leucoanthocyanidin reductase (LAR) (Dong and Lin, 2021). At the transcriptional level, R2R3-type MYB transcription factors play a conserved role in regulating flavonoid biosynthesis (Xu et al., 2015; Cao et al., 2021b). *CsMYB60* binds to the promoters of *CsFLS* and *CsLAR*, promoting flavonol and anthocyanidin biosynthesis in *Cucumis sativus* (Li et al., 2020b). The *MdMYB15L* interacts with *MdbHLH33* to suppress anthocyanin biosynthesis (Xu et al., 2018). In the Pear plant, *PyWRKY26* and *PybHLH3* interact and activate *PyMYB114* to promote anthocyanin biosynthesis (Li et al., 2020a). In plants, BRI1-EMS-SUPPRESSOR 1 (BES1), positively regulates brassinosteroids signaling and inhibits *MYB11*, *MYB12*, and *MYB111* resulting in decreased flavonol formation (Liang et al., 2020).

Flavonoids regulate the coloring of tissues and organs such as flowers, fruits, seeds, and also play a crucial role in reproduction, including seed formation and pollen fertility (Martínez-Silvestre et al., 2022; Qiao et al., 2022; Gang et al., 2019). In *B. platyphylla*, previous studies have isolated phenylbutanoids, lignans, triterpenoids, diarylheptanoids, phenolics and flavonoids (Rastogi et al., 2015). Additionally, the availability of chromosome-level genome and gene resources has enhanced the investigation of genes and pathways regulating growth and development (Chen et al., 2021). In this study, metabolites were isolated from different stages of staminate catkin and mature pollen. Subsequently, differential metabolite analysis was conducted to identify key metabolites regulating staminate catkin development and pollen maturation. Moreover, integrated transcriptomic and metabolomic analyses showed accumulation of flavonoid metabolites and key genes in the flavonoid pathway. Furthermore, identified key MYB genes showed a high correlation with quercetin derivatives, suggesting their potential roles in the flavonoid pathway. This research reveals novel insights about the flavonoids role in staminate catkin development and pollen maturation, suggesting their importance in plant reproduction.

## 2 Materials and methods

### 2.1 Plant materials

Flowers with staminate catkin and mature pollen were collected from *B. platyphylla* trees located in Harbin City, Heilongjiang Province, at the Northeast Forestry University. The developmental stages of the staminate catkins were categorized into the following: Stage 1 (S1), interconnected microsporocytes; Stage 2 (S2), uninuclear microspores undergoing differentiation and division; Stage 3 (S3), uninuclear microspores with three pores; Stage 4 (S4), bicellular microspores; and Stage 5 (S5), mature pollen (Zhang et al., 2023a).

### 2.2 Sample collection and metabolite isolation

The flowers were collected and promptly freezedried in a vacuum freezedryer (Scientz-100F). After freezedrying, the flower samples were grinded with zirconia beads for 1.5 minutes at 30 Hz in a mixer (MM 400, Retsch). Subsequently, 50 mg of grinded sample was mixed with 1.2 mL of a 70% methanol. The solution was vortexed 6 times, each lasting 30 seconds and occurring at 30-minute intervals. Afterward, the solution was centrifuged at 12000 rpm for 3 min. The resulting extracts were filtered through a 0.22 μm PTFE filter (SCAA-104, ANPEL, Shanghai, China) before being subjected to UPLC-ESI-MS/MS analysis.

### 2.3 Liquid chromatography and electrospray ionization mass spectrometry

The samples were subjected to UPLC analysis. UPLC separation was conducted using a Shimadzu Nexera X2 UPLC system. The mobile phase consisted of water with 0.1% methanoic (solvent A) and acetonitrile with 0.1% methanoic acid (solvent B). The gradient program was held for 1 minute with 95% A and 5% B, transitioning to 5% A and 95% B, followed by a return to 95% A and 5% B within 1.1 minutes, which was maintained for 2.9 minutes. The flow rate was 0.35 mL min⁻¹, and injection volume 4 µL. The column effluent was directed to a quadrupole-linear ion trap (QTRAP)-MS for detection, using an Applied Biosystems 4500 Q TRAP mass spectrometer.

### 2.4 Quadrupole linear ion mass spectrometry with electrospray ionization

The electrospray ionization source was operated at 550°C, with an ion spray voltage of 5500 V (positive) or -4500 V (negative). The gas pressures were set to 50, 60, and 25 psi (CUR). Collision-activated dissociation (CAD) was high. Calibration used 10 and 100 μmol L^-1^ polypropylene glycol solutions in QQQ and LIT modes. QQQ scans were acquired as MRM experiments with collision gas (nitrogen) set to medium. DP (declustering potential) and CE (collision energy) for individual MRM transitions was done with further DP and CE optimization. A specific set of MRM transitions were monitored for each period according to the metabolites eluted within this period.

### 2.5 Metabolomics analyses

The dendrograms, hierarchical cluster analysis (HCA), and Pearson Correlation Coefficients (PCC) between samples of samples were generated using the ‘cor.test’ function in R and presented exclusively as heatmaps. The scale function in R was used to normalize and visualize the signal of metabolites as a color spectrum. The compound and pathway databases (http://www.kegg.jp/kegg/compound/) (http://www.kegg.jp/kegg/pathway.html) were used for the identification, annotation, and mapping of metabolites. The analyst version 1.6.3, AB Sciex software was used for spectrometry data collection and validation. For two-group comparisons analysis, Variable Importance in Projection (VIP ≥ 1) and absolute Log_2_ fold change (|Log_2_FC| ≥ 1.0) isolated differential metabolites (Zhang et al., 2024a). Flavonoids were extracted and subjected to K-means clustering to group metabolites with similar profiles. The optimal number of clusters was obtained by using the within-cluster sum of squares (WSS) method, and clustering was performed using the “K-means” function in R. The R package “Mfuzz” was used to cluster the expression patterns of transcriptomic data and flavonoids.

### 2.6 Weighted gene co-expression network analysis

The transcriptome data (Zhang et al., 2023a) was used to determine the relationship between metabolites and gene co-expression through WGCNA. To evaluate the association between each metabolite and the WGCNA modules, the PCC and their associated *P* value were queried using the ‘cor.test’ function in R. Metabolites with significant correlations (0 < *P* value < 0.05 and correlation coefficient > 0.5) were selected for further analysis. The co-expression networks were identified in the MEblue module genes and interaction networks were visualized using the cytoscape software.

### 2.7 MiRNA target prediction

The miRNA targets were predicted using default parameters at the plant small RNA Target analysis server (psRNATarget server) (https://www.zhaolab.org/psRNATarget/), and cytoscape software was used to visualize the data.

### 2.8 GO enrichment analysis

The GO enrichment analysis of MEblue module genes was conducted with the clusterProfiler R package, in which the gene length bias was corrected. Significantly enriched GO terms were identified based on a corrected *P* value threshold of < 0.05.

### 2.9 RNA isolation and qRT-PCR

Samples were flashfrozen and instantly kept at -80°C using liquid nitrogen. The Total RNA was isolated using the Pure Plant Kit RNAprep (DP441, Tiangen, China) and RNA quality was assessed using NanoPhotometer (Implen). cDNA was synthesised with the One-Step gDNA Removal and cDNA Synthesis SuperMix (Transgen, Switzerland), followed by qRT-PCR using TransStart^®^ Top Green qPCR SuperMix (Transgen, Switzerland). The experiment was conducted with three biological replicates. *BpTUB* (*BpChr11G09309*) was selected as an internal control, and gene expression was determined by the 2^−ΔΔCT^ method. The Primer-Blast tool was used to design qPCR primers, which are listed in **Supplementary Table S4**.

## 3 Results

### 3.1 Characterization of metabolite profiles during staminate catkin development and pollen maturation in *B. platyphylla*

**Figure 1:**
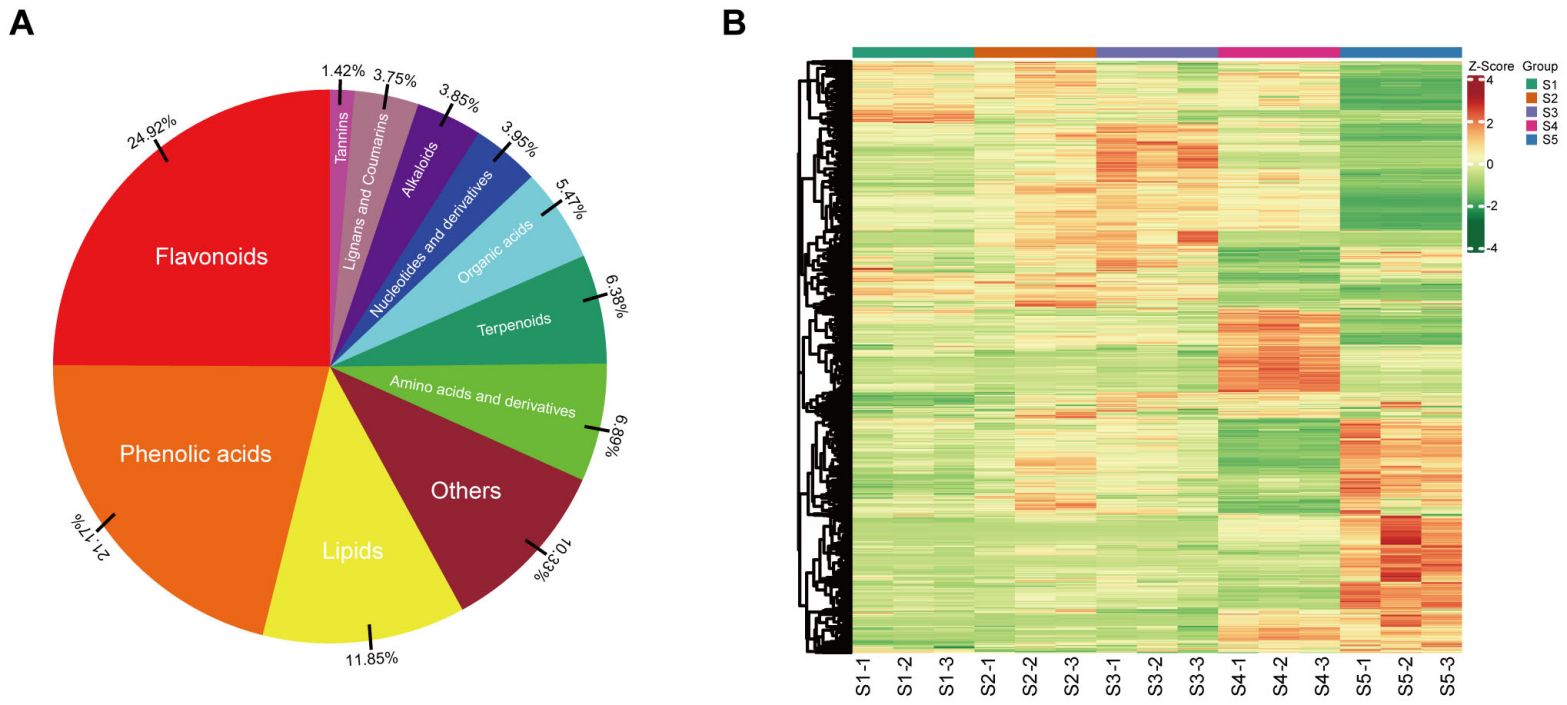
Comprehensive profiling of the metabolites during staminate catkin development and pollen maturation in *B. platyphylla*. **(A)** Classification of the 987 metabolites into 11 metabolite classes; **(B)** Heatmap showing hierarchical clustering of all detected metabolites at different developmental stages of staminate catkin and pollen maturation. Columns represent sample stages, and metabolites are represented by rows. Red color denote relatively high metabolite abundance, and green colors denote relatively low abundance.

An extensive targeted metabolomics study was conducted using UPLC-ESI-MS/MS to gain insight into the metabolic features of staminate catkin stages and pollen maturation in *B. platyphylla.* In this study, the staminate catkin was divided into four stages: interconnected microsporocytes as S1, uninuclear microspores differentiated and divided as S2, uninuclear microspores with three pores as S3, bicellular microspores as S4 and mature pollen as S5. We identified a total of 987 metabolites across 11 classes, including 246 flavonoids, 209 phenolic acids, 117 lipids, 68 amino acids and derivatives, 63 terpenoids, 54 organic acids, 39 nucleotides and derivatives, 38 alkaloids, 37 lignans and coumarins, and 14 tannins, accounting for 24.92%, 21.17%, 11.85%, 6.89%, 6.38%, 5.47%, 3.95%, 3.85%, 3.75% and 1.42%, respectively, (**Figure 1A**; **Supplementary Figure S1**). Hierarchical cluster analysis (HCA) revealed distinct patterns of metabolite accumulation from S1 to S5 of *B. platyphylla*. Specifically, samples S2 and S3 appear to cluster more closely together, showing similar metabolic patterns. In contrast, S1, S4, and S5 seem to form another distinct cluster, indicating different metabolic profiles. Furthermore, the metabolites content and composition were most enriched in stage S5 (**Figure 1B**). These results suggest that metabolites vary among tissues and are stage-specific during the *B. platyphylla* staminate catkin development and pollen maturation.

### 3.2 Differential metabolite analysis across stages of staminate catkin and pollen maturation in *B. platyphylla*

**Figure 2:**
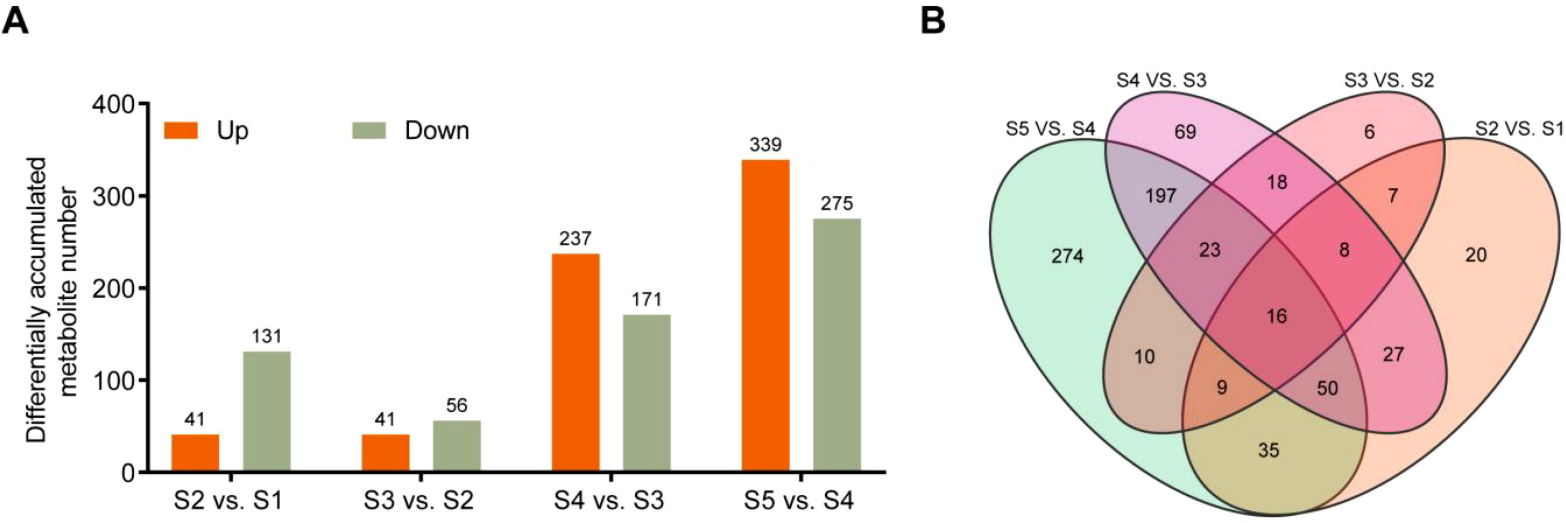
Differential Metabolites (DMs) were analyzed in the *B. platyphylla* staminate catkin and mature pollen. **(A)** The number of DMs and **(B)** Venn diagrams showing the overlap of different metabolites in multiple pairwise comparisons in group of S2 *vs*. S1, S3 *vs*. S2, S4 *vs*. S3 and S5 *vs*. S4.

To further investigate how metabolites accumulate during the transition from staminate catkin stages to mature pollen in *B. platyphylla*, the Variable Importance in Projection (VIP) and fold change (FC) were combined to further screen out differential metabolites (DMs). The comparisons between S2 *vs*. S1, S3 *vs*. S2, S4 *vs*. S3, and S5 *vs*. S4 were analyzed, and the metabolites with thresholds of VIP ≥ 1 and |log_2_FC| ≥ 1.0 were identified as DMs. Of them, 41, 41, 237, and 339 metabolites showed higher abundance, and 131, 56, 171, and 275 metabolites showed lower abundance in the comparisons of S2 *vs*. S1, S3 *vs*. S2, S4 *vs*. S3 and S5 *vs*. S4 respectively (**Figure 2A**). The Venn diagram revealed a distribution of overlapped or stage-specific metabolites, showing dynamic changes in each group. We identified 16 DMs accumulated in all comparison groups, with flavonoids being the most abundant class (**Figure 2B**; **Supplementary Table S1**). The most abundant metabolites in the four comparison groups were flavonoids (**Supplementary Figure S2**), suggesting that flavonoid accumulation may play a vital role in staminate catkin development and pollen maturation in *B. platyphylla*. Therefore, downstream analysis focused on the flavonoids.

### 3.3 Clustering analysis revealed a stage-specific dynamic accumulation pattern of flavonoids during staminate catkin development and pollen maturation in *B. platyphylla*.

**Figure 3:**
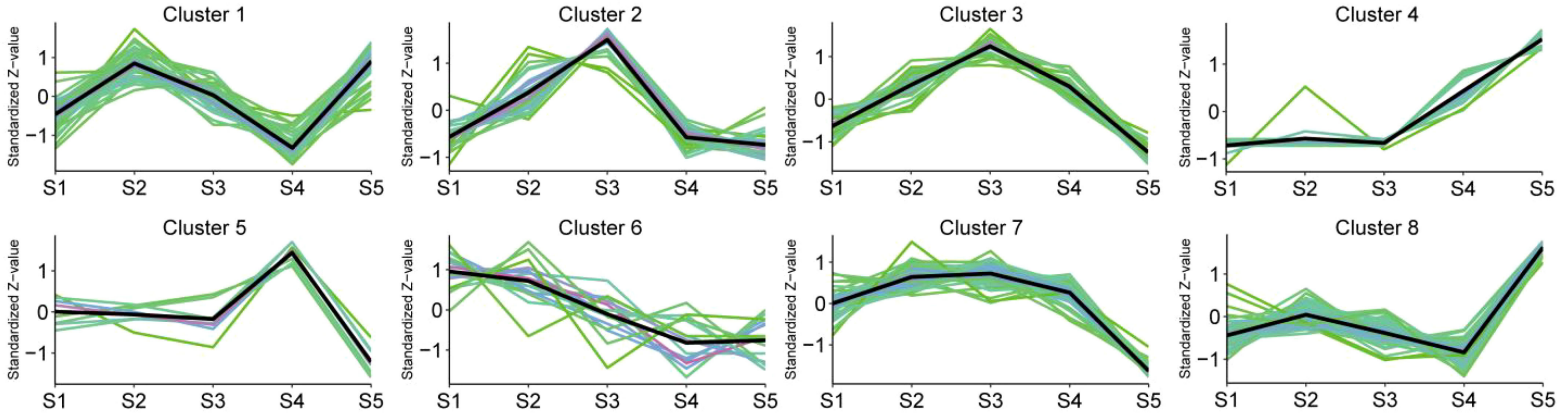
K-means clustering analysis of 246 flavonoid metabolites according to their variation tendencies.

**Figure 4:**
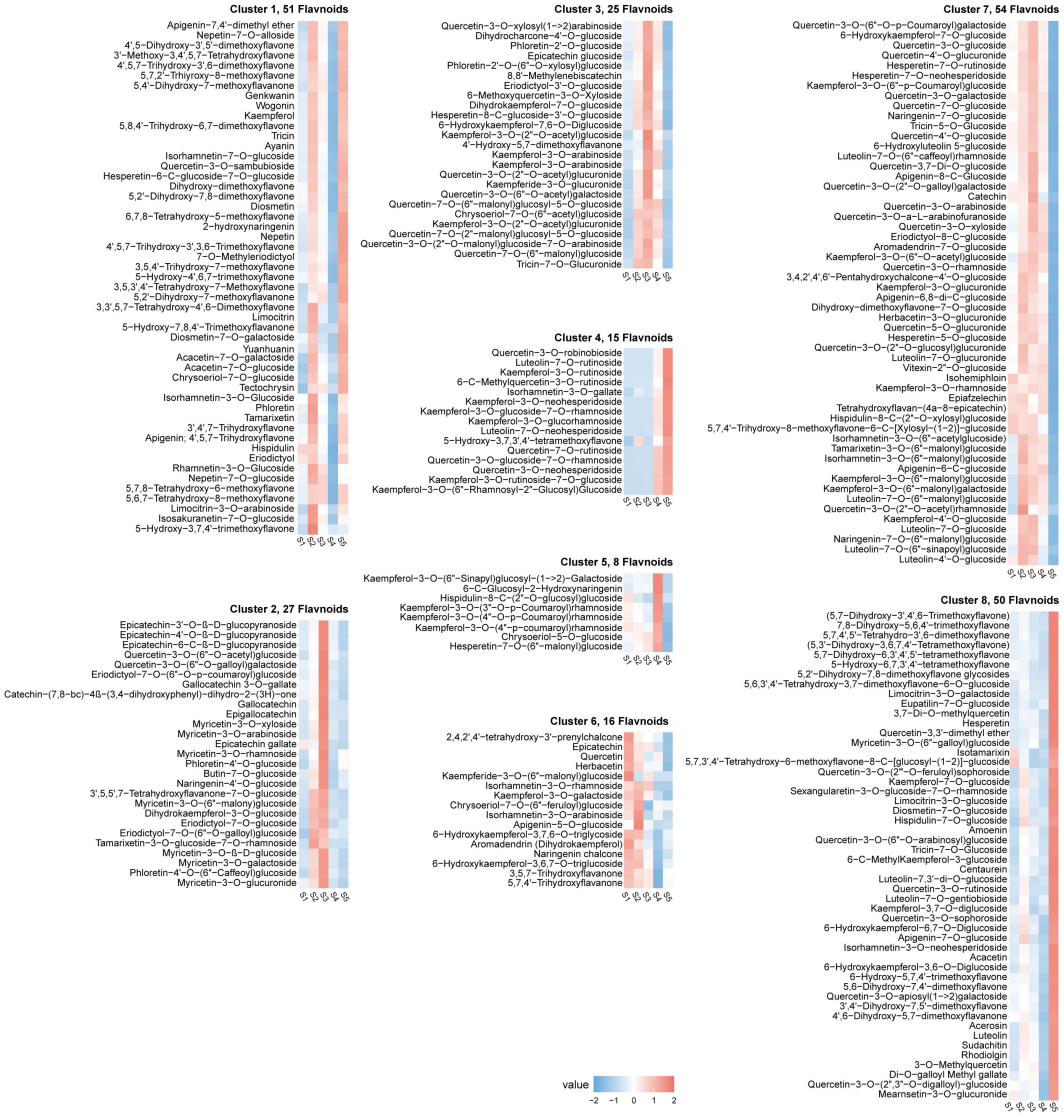
Dynamics of flavonoid metabolites in eight clusters.

The algorithm of K-means and Mfuzz clustering were used to investigate the role of flavonoids in staminate catkin development and pollen maturation in *B. platyphylla*. Two hundred and fourty six (246) flavonoids metabolites were identified and grouped into eight distinct clusters, revealing potential functional differences among them (**Figure 3**; **Supplementary Figure S3A**). These clusters revealed stage-specific accumulation patterns for each flavonoid, highlighting their dynamic changes across developmental stages. Flavonoids metabolites in K-means Cluster 1, comprising 51 flavonoids, exhibited predominant accumulation during the S2 and S5 stages. Clusters 2 and 3, containing 27 and 25 flavonoids respectively, demonstrated higher abundance during the S3 stage. Clusters 4 and 8, consisting of 15 and 50 flavonoids, had minimal accumulation from S1 to S4 but displayed specific accumulation during the S5 stage. Cluster 6, which included 16 flavonoids, was highly abundant in the early stages of staminate catkin development (S1 and S2). In contrast, clusters 5 and 7, encompassing 8 and 54 flavonoids respectively, exhibited reduced accumulation during the S5 stage (**Figure 4**). Furthermore, using the Mfuzz software, time-series clustering analysis of transcriptomic data revealed eight clusters (C1 to C8), and cluster 4 and 8 exhibited the same expression patterns as flavonoids metabolites, and showed the highest accumulation in the S5 stage (**Supplementary Figure S3B**). This clustering analysis highlights distinct stage-specific accumulation patterns of flavonoids and genes clusters during staminate catkin development and pollen maturation in *B. platyphylla*. The “co-abundance” characteristics in each cluster reveal that genes and metabolites are correlated in their regulation across the S1 to S5 stages of catkin and pollen development.

### 3.4 WGCNA-based analysis of flavonoid biosynthesis genes at staminate catkin development and pollen maturation in *B. platyphylla*

**Figure 5:**
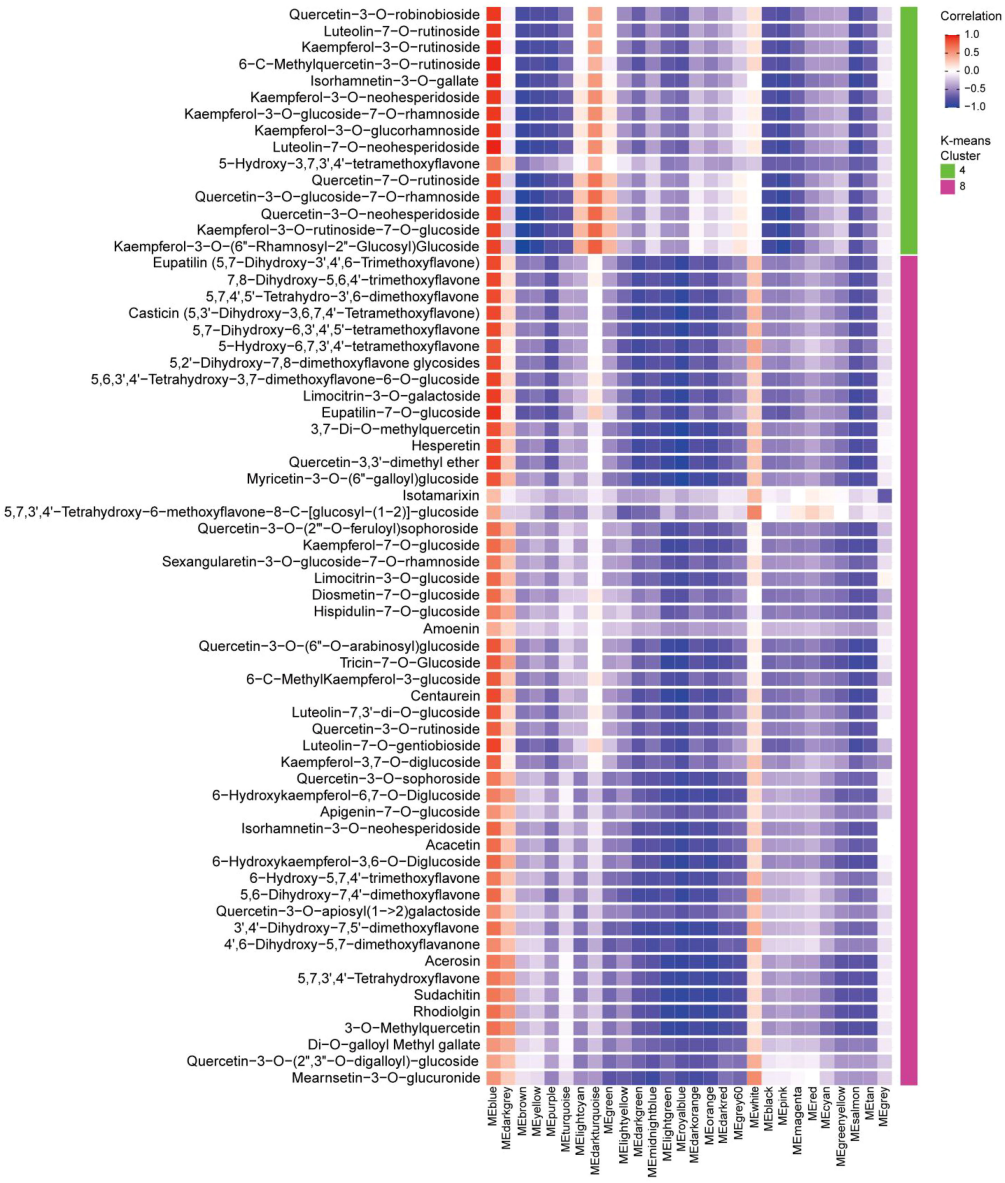
Exploring the correlation between gene modules and flavonoids. The correlation coefficients were calculated between 65 flavonoids (specifically accumulated during the S5 stage in clusters 4 and 8) and the 28 gene modules. Red color indicates positive correlations, while blue color represents negative correlations. The gene modules is plotted on X-axis, and the flavonoid compounds were plotted on the Y-axis. This visualization highlights the relationship between genes and flavonoids during staminate catkin development and pollen maturation in *B. platyphylla*.

To reveal correlation of gene expression and flavonoid contents, eight clusters were first identified through K-means clustering. Subsequently, WGCNA was performed for key gene modules identification. This analysis identified 28 unique modules, each displaying distinct correlation patterns with the eight flavonoid clusters (**Supplementary Figure S4**). The MEblue module showed a strong positive correlation with 65 flavonoids metabolites (*P* value < 0.05 and correlation coefficient > 0.5), which exhibited the highest specificity and accumulation at the S5 stage (**Figure 5**). However, only 10 of these flavonoids were enriched in the KEGG pathway analysis (**Supplementary Table S2**). Among these, four quercetin derivatives were identified: 3-O-Methylquercetin, 3,7-O-Dimethylquercetin, Quercetin-3-O-rutinoside and Quercetin-3-O-sophoroside. These results strongly suggest that quercetin derivatives play a crucial role in pollen maturation of *B. platyphylla*.

### 3.5 Integrated flavonoid biosynthesis pathway analysis in *B. platyphylla* staminate catkins

To explore the metabolites and key genes associated with the flavonoid biosynthesis pathway, an integrated transcriptomic and metabolomic analysis was conducted. The results revealed the top 25 co-enriched KEGG pathways across four comparison groups: S2 *vs*. S1, S3 *vs*. S2, S4 *vs*. S1, and S5 *vs*. S4 (**Supplementary Figure S5**). Key metabolites within the flavonoid biosynthesis pathway, such as phenylalanine, cinnamic acid, naringenin chalcone, naringenin, dihydrokaempferol, and quercetin, were identified across all four groups. Additionally, early acting structural genes involved in this pathway, including *Bp4CL*, *BpCHI*, three *BpCHS* isoforms, *BpF3H*, *BpCYP75A*, *BpCYP75B1*, three *BpFLS* isoforms, and three *BpPAL* isoforms, were also identified in these comparisons. A strong correlation between metabolites and structural genes within the flavonoid biosynthesis pathway was observed. Flavonoid metabolites and structural genes with significant accumulation during the early stages, particularly noted in the S1 stage of staminate catkin development.

**Figure 6:**
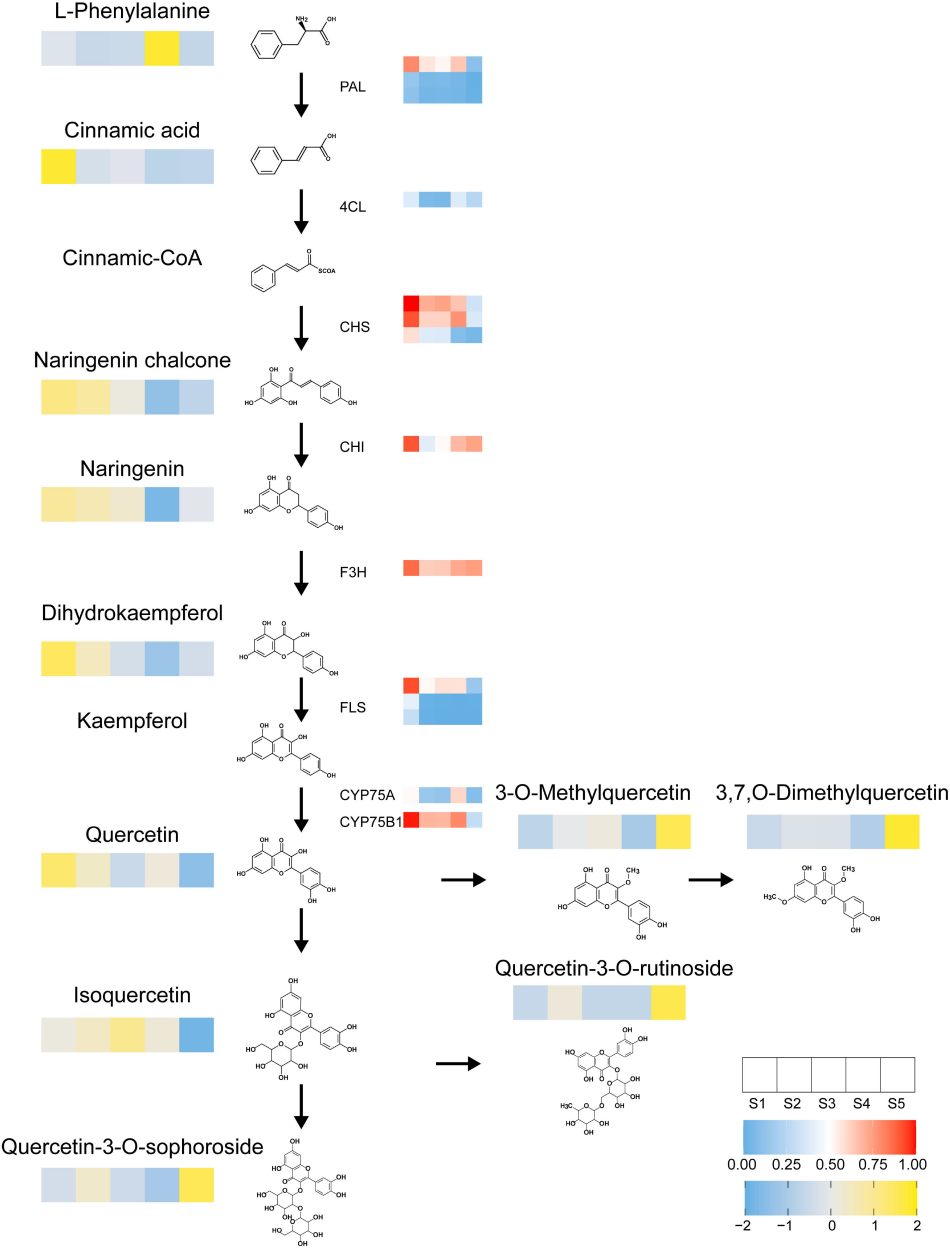
Heatmap illustrates the flavonoid biosynthesis pathway. Depicting the expression profiles of metabolites and genes across different developmental stages of staminate catkin and mature pollen in *B. platyphylla*. Each row represents a flavonoid metabolite or gene, with colors indicating relative abundance or expression levels. Yellow denotes high-abundance flavonoids, while blue signifies low-abundance flavonoids. Similarly, red denotes high gene expression levels, and blue denotes low gene expression levels. The heatmap provides a comprehensive visualization of stage-specific variations in flavonoid metabolites and gene regulation during development.

Furthermore, we selected MEblue module genes related to the metabolism of flavonoid metabolites and the top 20 flavonoid metabolites, including two quercetin derivatives (Lmsp004166, Lmjp002461), two naringenin derivatives (pme0376, pme2960), and three kaempferol derivatives (mws1068, Xmyp004945, Lmpp003268), and a significant correlation was observed (**Supplementary Figure S6A**). However, although it serves as the primary transcriptional regulator of flavonoid biosynthesis, MYB family genes were not found in the top 20 (**Supplementary Figure S6B**), suggesting that additional factors or regulatory mechanisms may be involved. These findings suggest that different components of the integrated model capture distinct aspects of the transcriptional and metabolic responses, potentially reflecting the underlying more biological processes and pathways involved during staminate catkin development and pollen maturation. Based on the identified flavonoid metabolites and structural genes, we identified the key pathways regulating flavonoid biosynthesis (**Figure 6**).

### 3.6 Identification and expression analysis of candidate genes associated with quercetin derivatives in the flavonoid biosynthesis pathway

The MYB-bHLH-WD repeat (MBW) complex, comprised of MYB, bHLH, and WD40 proteins as primary transcriptional regulators of flavonoid biosynthesis (Xu et al., 2015). The MEblue module detected 134 transcription factors (TFs) from various families, including MYB TFs. Subsequent GO annotation showed these genes to be enriched in biological processes related to localization and transport, cellular components including intracellular anatomical structures and cytoplasm, and molecular functions such as protein binding and catalytic activity (**Supplementary Figure S7**).

Moreover, domain analysis identified eight MYB transcription factors (TFs). Including *BpMYB18* (*BpChr03G09649*), *BpMYB42* (*BpChr06G22567*), *BpMYB72* (*BpChr11G05659*), *BpMYB95* (*BpChr11G28685*), *BpMYB109* (*BpChr13G10264*), *BpMYB112* (*BpChr14G01120*), and *BpMYB113* (*BpChr14G01859*). Each of which contains two MYB DNA-binding domains, thereby categorizing them as R2R3-MYB family members (**Supplementary Figures S8A, B**). Notably, R2R3-MYB TFs can recognize MYB cis-elements in flavonoid biosynthesis gene promoters to regulate their transcription (Wang et al., 2019b). Promoter cis-elements analysis of the flavonoid biosynthesis genes revealed that they contain MYB cis-elements in their promoters (**Supplementary Table S3**), suggesting a potential regulation of these genes by MYB TFs during flavonoid biosynthesis.

**Figure 7:**
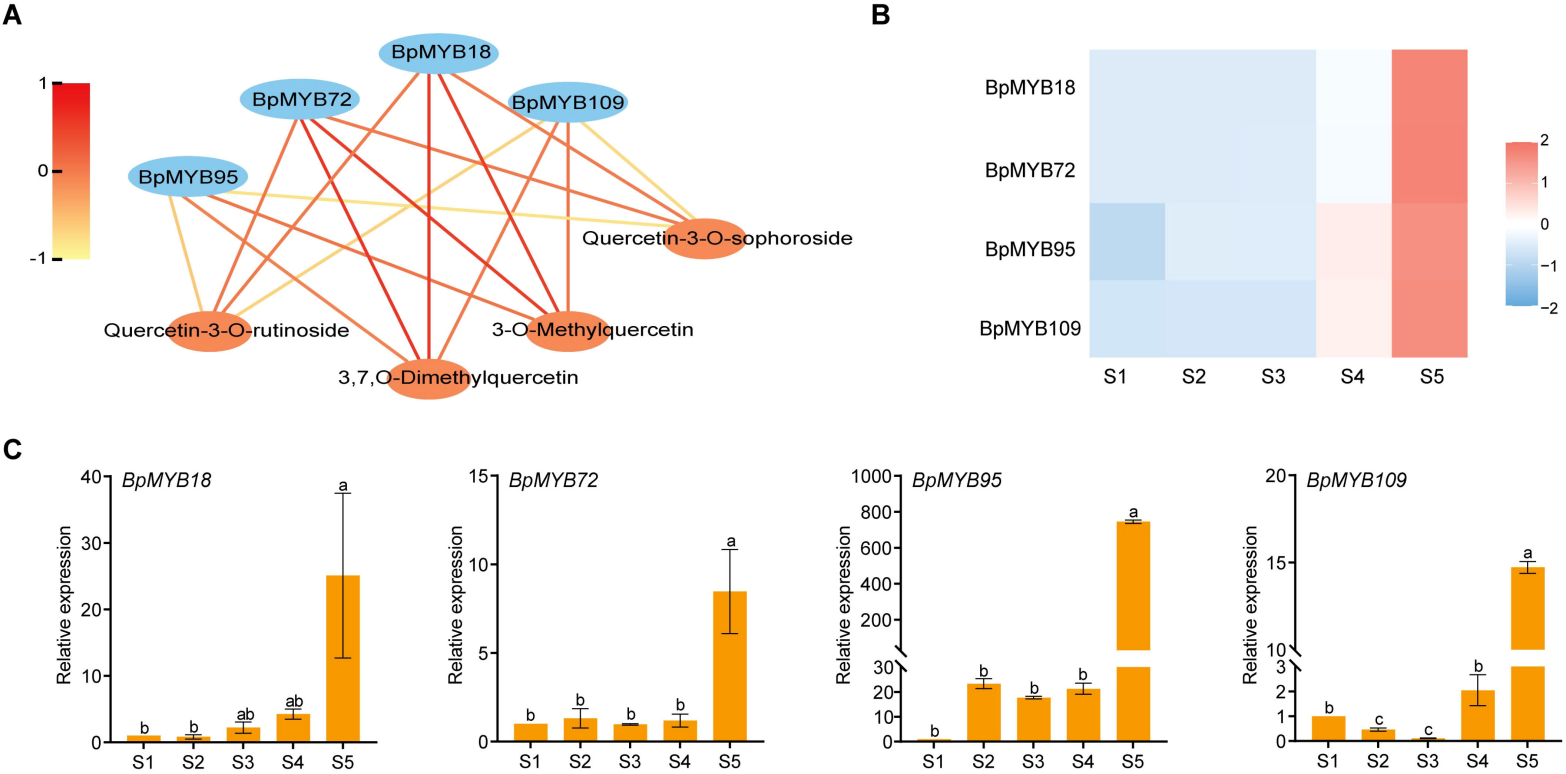
Correlation and expression analysis of MYB genes. **(A)** Correlation analysis between *BpMYB18*, *BpMYB72*, *BpMYB95*, *BpMYB109*, and quercetin derivatives, highlighting their potential regulatory relationships. Red line denotes a strong association, while yellow signifies a weak association. **(B)** Expression patterns of the four MYB genes derived from RNA-Seq data across different developmental stages. **(C)** RT-qPCR validation of the expression levels of the MYB genes during staminate catkin and mature pollen development in *B. platyphylla*. Letters on the bars denote statistical significance (*P*<0.05) as obtained by one-way ANOVA (LSD-Duncan method).

Integration of WGCNA data revealed strong correlations between specific *MYB* genes (*BpMYB18*, *BpMYB72*, *BpMYB95*, and *BpMYB109*) and quercetin derivatives (3-O-Methylquercetin, 3,7-O-Dimethylquercetin, Quercetin-3-O-rutinoside, and Quercetin-3-O-sophoroside). RNA-Seq analysis revealed significant expression of these *MYB* genes in mature pollen, with expression patterns coinciding with the accumulation of corresponding quercetin derivatives at stage S5 (**Figures 7A–C**). We identified *BpChr04G24530*, *BpChr03G01033*, *BpChr07G18900*, and *BpChr11G10134* in a purple box, showing the common points of interaction among four *BpMYBs* (*BpMYB18*, *BpMYB72*, *BpMYB95*, and *BpMYB109*), which play a role in reproductive development (**Supplementary Figure S9A**). Furthermore, we discovered that three miR319 and three miR159 family members (yellow box) potentially regulate *BpMYB72*, *BpMYB95*, and *BpMYB109* through post-transcriptional silencing (**Supplementary Figure S9B**). Collectively, these results strongly suggest that *BpMYB18*, *BpMYB72*, *BpMYB95*, and *BpMYB109* regulate the biosynthesis of quercetin derivatives.

## 4 Discussion

Plants can synthesize a wide variety of secondary metabolites to adapt to myriad environmental stimuli (Kessler and Kalske, 2018). Based on their chemical structure, plant secondary metabolites comprise phenolic acids, flavonoids, terpenoids, steroids, and alkaloids, all of which are crucial for plant growth and development (Yang et al., 2018). Flavonoids are derived from polyphenolic compounds biosynthesized as secondary metabolites in plants (Zhao et al., 2023). Flavonoids can exhibit various functions that impact plant growth, including reactive oxygen species (ROS) scavenging ability (Cavaiuolo et al., 2013), auxin metabolism (Tan et al., 2019), growth regulation (Thitz et al., 2021), biotic and abiotic stress mitigation (Bidel et al., 2007), temperature stress response (He et al., 2023; Zhang et al., 2023b). Further, they are potential medicines for disease treatment (Ullah et al., 2020). *B. platyphyla* possesses various pharmacological properties, for example, *B. platyphyla* leaf extracts demonstrate antioxidant, antibacterial, and anticancer activity (Acquaviva et al., 2013; Penkov et al., 2018; Ju et al., 2004). Earlier research indicated that flavonoids isolated from the buds of *B. platyphyla* possess anti-cancer activity (Szoka et al., 2021). Moreover, flavonoid compounds, which are present in flowers, fruits, leaves, and seeds, have an impact on pollen bioactivity (Kerienė et al., 2023a). Pollen releases phenolic compounds and provides a nutrient source for aquaculture when dispersed on surface waters (Kerienė et al., 2023b). Similarly, compounds and end products of the flavonoid pathway confer anti-herbivore functions on *B. platyphyla* (Thitz et al., 2020). Flavonoids from *B. platyphyla* buds exhibited strong biological activity and defensive properties (Szoka et al., 2021; Isidorov et al., 2022). However, there are few studies on the role of flavonoids in the context of the staminate catkin development and pollen maturation in *B. platyphyla*.

In our previous study, the staminate catkin of *B. platyphylla* was exposed to a prolonged low temperature period during development (Zhang et al., 2023a). Low temperature and total flavonoid may have a significant impact on staminate catkin development (He et al., 2023). To investigate presence of key metabolites and metabolic pathways during the developmental stages of staminate catkin and pollen maturation, metabolite abundance was analyzed at four different stages of staminate catkin development and pollen maturation in *B. platyphylla.* Flavonoids were the most abundant metabolites detected, accounting for 24.92% of the total metabolite content and significantly accumulating in mature pollen (**Figure 1**). Previous research shows that flavonoids are essential for male reproductive development (Woo et al., 2005; Gong et al., 2024; Sun et al., 2022). Metabolite content and composition show dynamic changes in different tissues and stages, and our data implies that flavonoids are possibly major players in the process of staminate catkin development and pollen maturation.

Based on the Venn diagram analysis, 16 metabolites were determined to be “core metabolites” across various stages of the staminate catkin development and pollen maturation (**Figures 2A, B**). Also, the KEGG analysis showed that among the 16 metabolites identified, 50% of the metabolites showed higher enrichment in flavonol and flavonoid biosynthesis pathways (ko00944 and ko00941) (**Supplementary Table S1**). It is worthy to note that integrated transcriptome and metabolome analysis serve as a powerful tool to study different aspect of plant physiology and development (Wang et al., 2022). Flavonoids have been found to modulate wheat response to powdery mildew using integrated metabolomics and transcriptomic analyses (Xu et al., 2023). In the current study, K-means analyses revealed two clusters of the flavonoid metabolites that showed high accumulation in mature pollen (**Figures 3**, **4**). Combined with WGCNA result, MEblue module showed strongest positive correlation with flavonoids accumulated in mature pollen (**Figure 5**). In addition, four quercetin derivatives were identified and enriched in the flavonoid and flavonol biosynthesis pathway (ko00944). These results indicate that the flavonoid pathway, particularly quercetin derivatives, plays a crucial role in staminate catkin development and pollen maturation in *B. platyphylla*.

Flavonoid biosynthesis and its pathway regulation have been well studied in model plants (Hichri et al., 2011; Naik et al., 2022). Flavonoids in flowering plants are known to regulate pigmentation, thereby facilitating successful pollination (Falcone Ferreyra et al., 2012). In tobacco, substantial evidence indicates that flavonoids regulate flower development (Pattanaik et al., 2010). Moreover, various TFs were reported to be involved in the flavonoid pathway to regulate plant development (Nabavi et al., 2020; Winkel-Shirley, 2001). In our study, we identified the major metabolites and genes associated with the flavonoid pathway. These metabolites and genes were found to accumulate at the early stage of staminate catkin development, while quercetin derivatives accumulated at the mature pollen stage (**Figures 5**, **6**). The MYB TF family is among the most widely distributed and the largest TF families in plants, playing crucial roles in diverse biological processes (Li et al., 2015). R2R3-MYB was reported to regulate flavonoid biosynthesis (Cao et al., 2024; Nabavi et al., 2020). *MtMYB134* can activate the promoters of *MtCHS2* and *MtFLS2*, and its overexpression in the hairy roots of *Medicago truncatula* enhances various flavonol derivatives biosynthesis (Naik et al., 2021). *PqMYBF1* activates the promoters of *PqCHS*, *PqF3H*, and *PqFLS*, thereby promoting flavonol biosynthesis in *Paeonia qiui* (Zhang et al., 2023c). The *MYB12* plays a key role in activating flavonol biosynthesis in response to light in *Arabidopsis* (Bhatia et al., 2021). According to our data, MYB genes in the MEblue module were identified through WGCNA. These genes showed a high correlation with quercetin derivatives and exhibited a similar accumulation pattern in mature pollen (**Figure 7**). Flavonoids are classified into several taxonomic category, and quercetin belongs to the flavonols subclass (Georgiou et al., 2023). Quercetin is a yellow-colored compound with multi-target bioactivity. However, its low solubility and poor bioavailability restrict its applicability (Alizadeh and Ebrahimzadeh, 2022; Bljajić et al., 2016). Thus, *BpMYB18*, *BpMYB72*, *BpMYB95*, and *BpMYB109* could promote quercetin derivatives biosynthesis in mature pollen, which is crucial for pollen maturation and plant reproduction.

## 5 Conclusion

In this study, we investigated the flavonoid pathway, which is a key pathway regulating staminate catkin development and pollen maturation in *B. platyphylla*, through comprehensive transcriptomic and metabolomic analysis. The metabolomic results showed that 246 flavonoid metabolites were identified with distinct stage-specific accumulation patterns during staminate catkin development and pollen maturation in *B. platyphylla.* Four quercetin derivatives including 3-O-Methylquercetin, Quercetin-3-O-rutinoside, 3,7-O-Dimethylquercetin, and Quercetin-3-O-sophoroside enriched in the flavonoid and flavonol biosynthesis pathway (ko00944) and specific accumulated in mature pollen. Combined metabolomic and transcriptomic analyses identified significant accumulation of key genes and major metabolites associated with the flavonoid pathway during the early stages of staminate catkin development, suggesting crucial roles in the initial development process. *BpMYB18*, *BpMYB72*, *BpMYB95*, and *BpMYB109* were found to be regulating flavonoid accumulation, indicating their crucial functions in the flavonoid pathway. Our findings provide the first comprehensive investigation of key metabolic pathways involved in staminate catkin development and pollen maturation in *B. platyphylla*. This research significantly enhances our knowledge of the mechanisms underlying reproductive development in this species and provides a valuable resource of potential genes for manipulating tree reproduction.

## Data Availability

The datasets presented in this study can be found in online repositories. The names of the repository/repositories and accession number(s) can be found below: https://www.ncbi.nlm.nih.gov/, PRJNA994611.
