# Supplementary material for: Integrated metabolome-transcriptome analyses reveal key pathways regulating staminate catkin development and pollen maturation in Betula platyphylla
Source: Front Plant Sci. 2025 May 21;16:1581560. doi: 10.3389/fpls.2025.1581560 (PMC12133839; doi:10.3389/fpls.2025.1581560)
Supplement: Supplementary file 1 [file DataSheet1.docx]

Supplementary Material


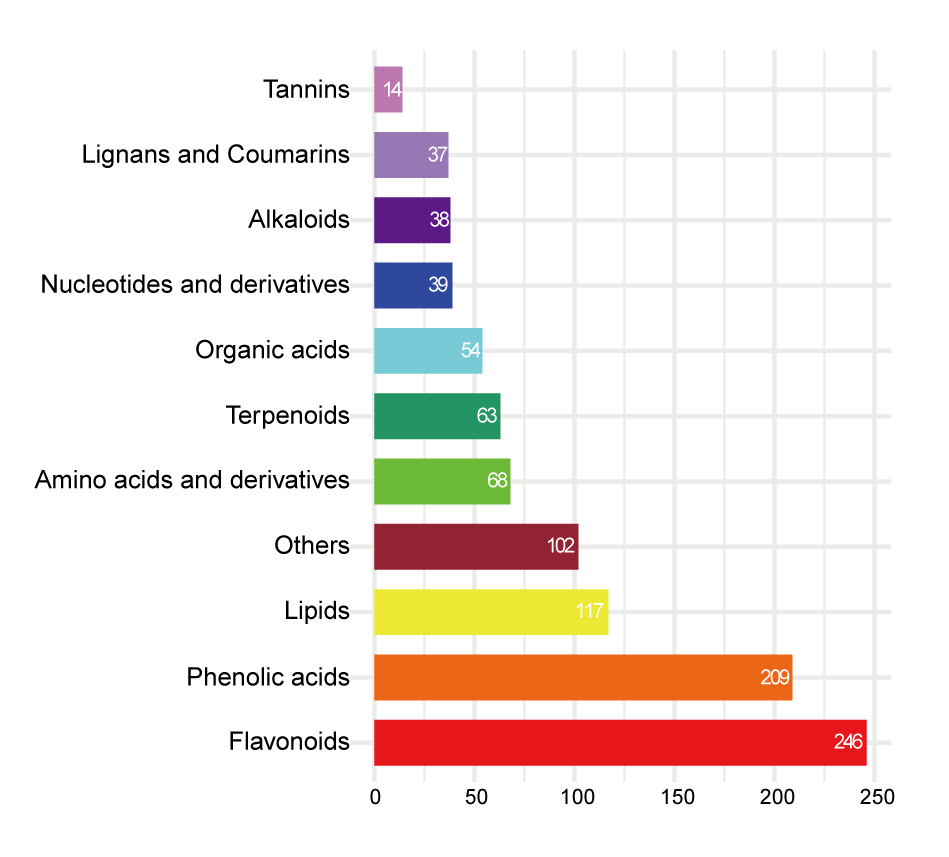


**Figure S1.** Number of identified metabolites classification histogram.


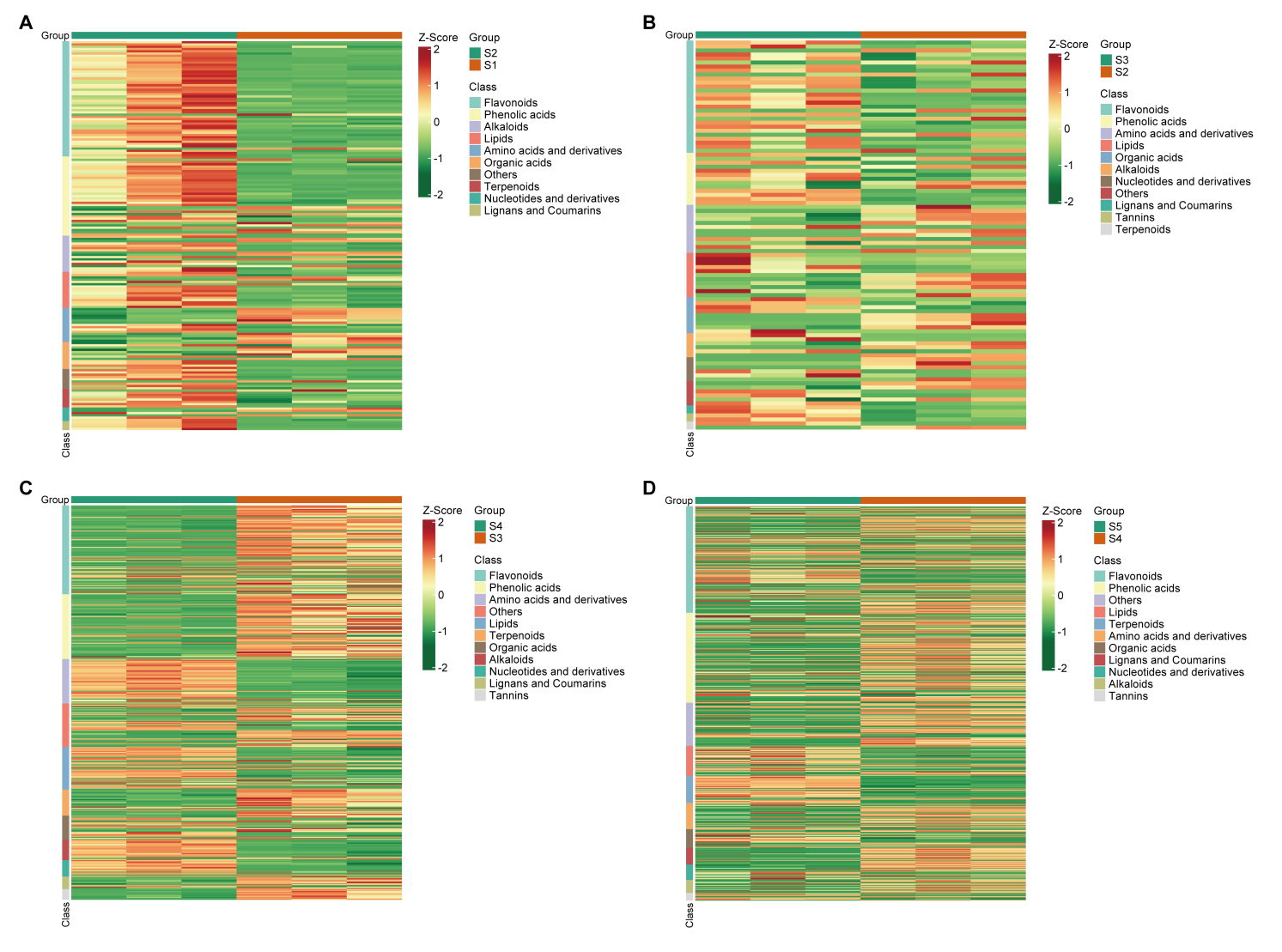


**Figure S2.** Hierarchical cluster analysis S2 vs. S1 (A), S3 vs. S3 (B), S4 vs. S3 (C), S5 vs. S4 (D). The ordinate shows the relative content of different class of metabolites. The green color indicates the low content, while the red color means high content.


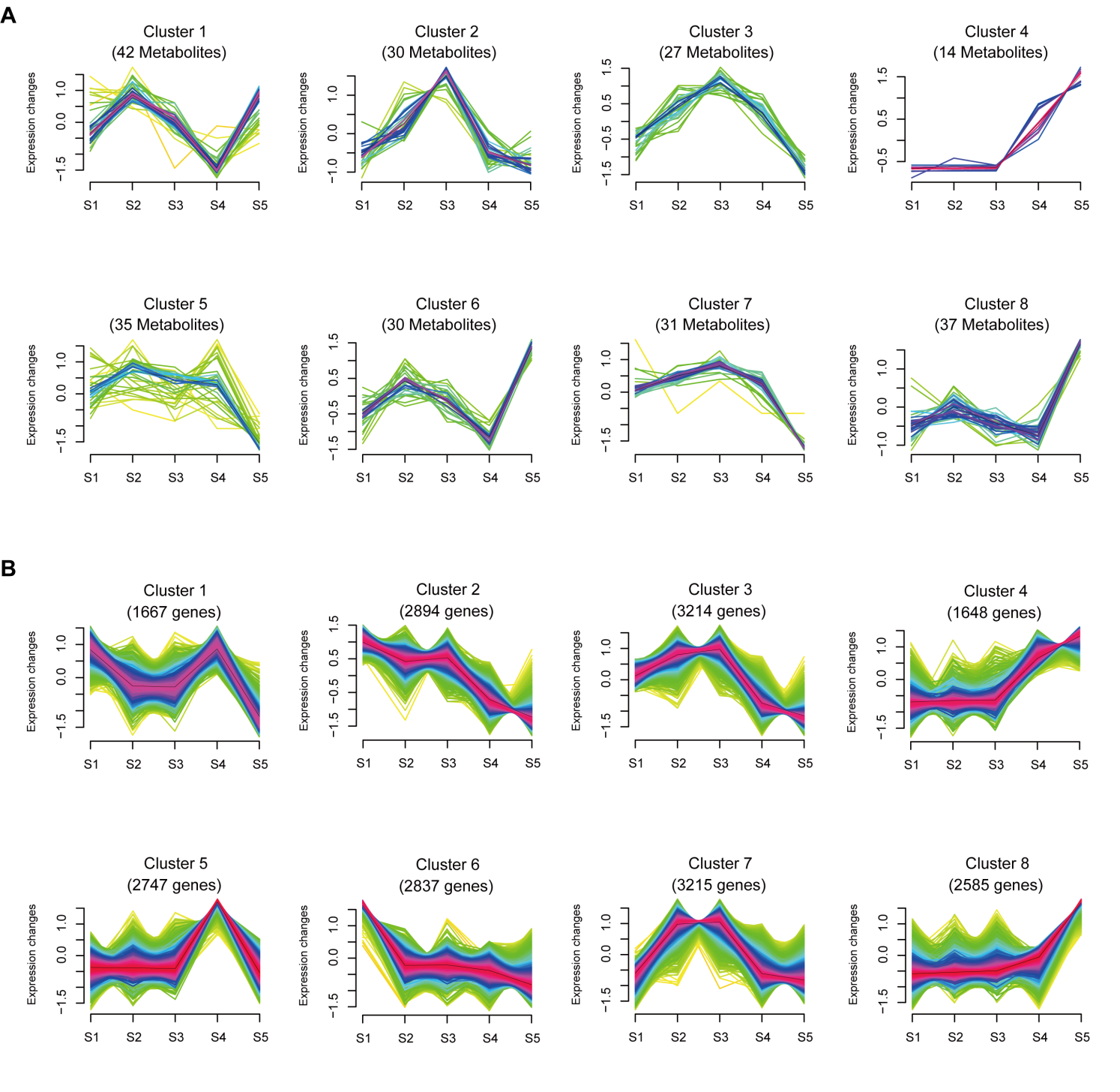


**Figure S3.** Mfuzz clustering in R of flavonoid metabolites (A) and transcriptomic data (B) that are temporally co-regulated across the S1 to S5 stages of staminate catkin development and pollen maturation.


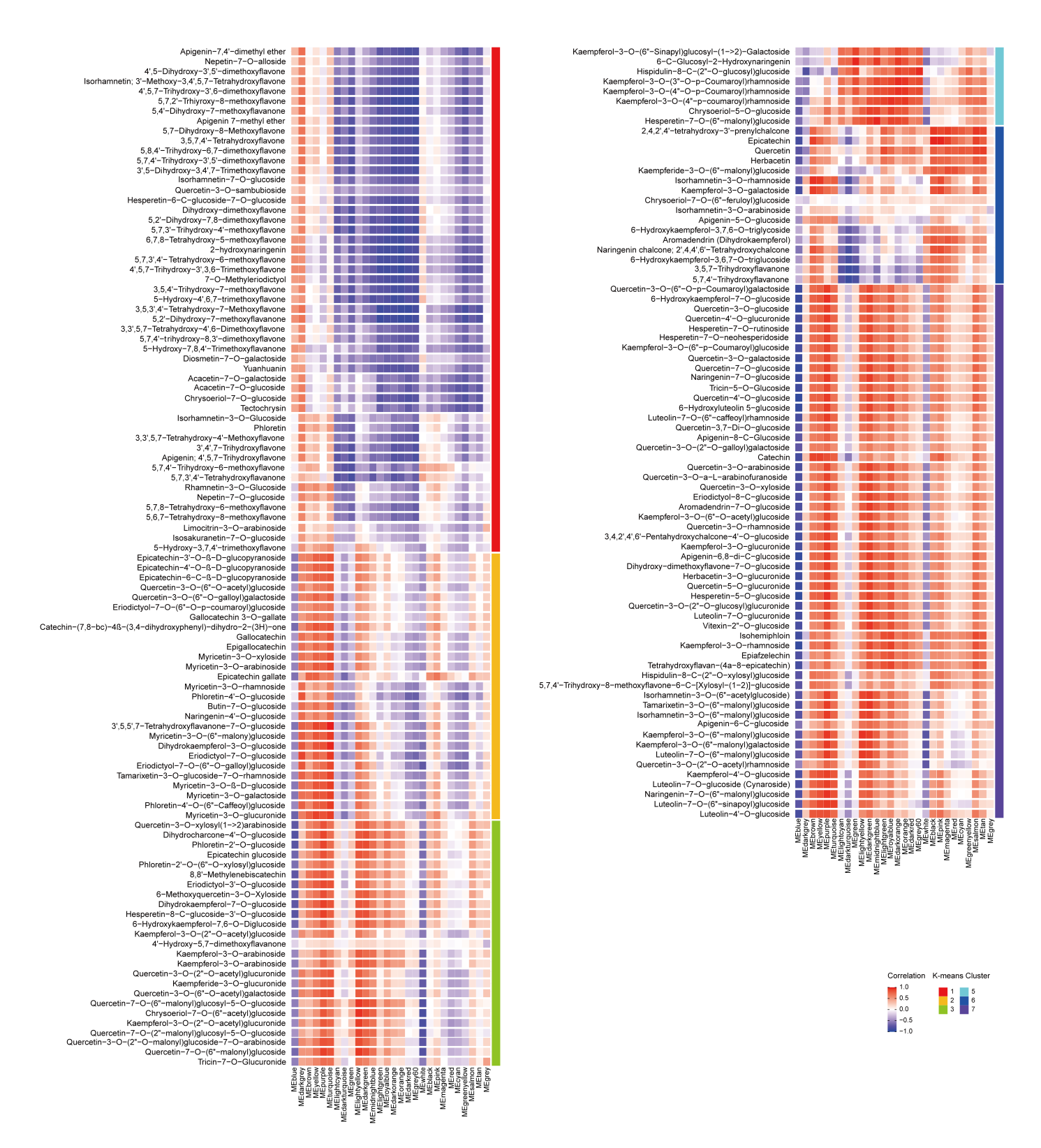


**Figure S4.** The results of WGCNA. Correlation coefficient between cluster 1, 2, 3, 5, 6, 7 with 28 module. Red indicates positive correlations, while blue represents negative correlations. The X-axis corresponds to the gene modules, and the Y-axis represents the flavonoid compounds.


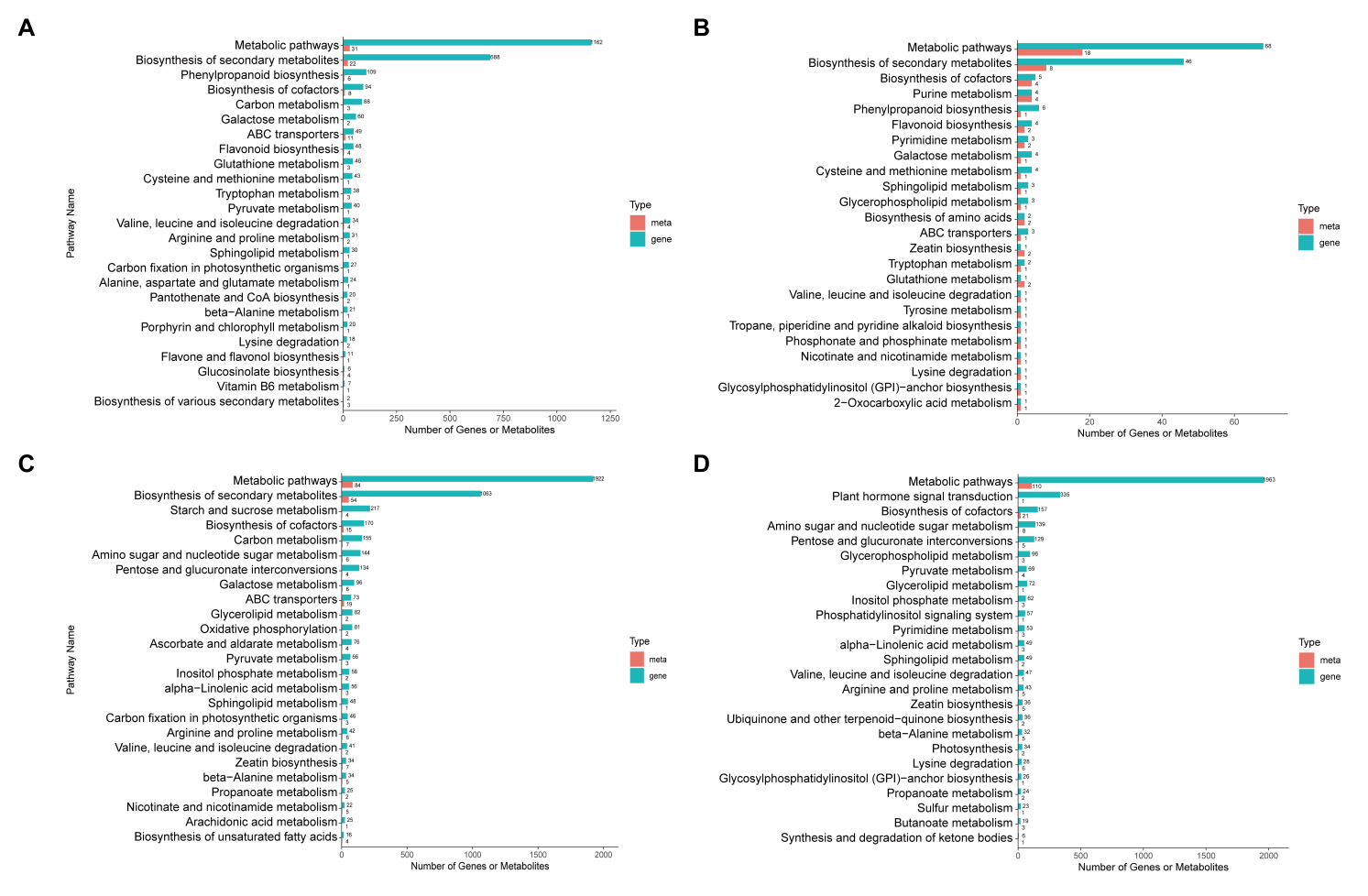


**Figure S5.** Top 25 KEGG co-enriched analysis combining transcriptome and metabolome. The X-axis represents the number of differential metabolites and differential genes enriched into the pathway, the Y-axis represents the KEGG pathway name, and the red and green bars represent the metabolome and transcriptome, respectively.


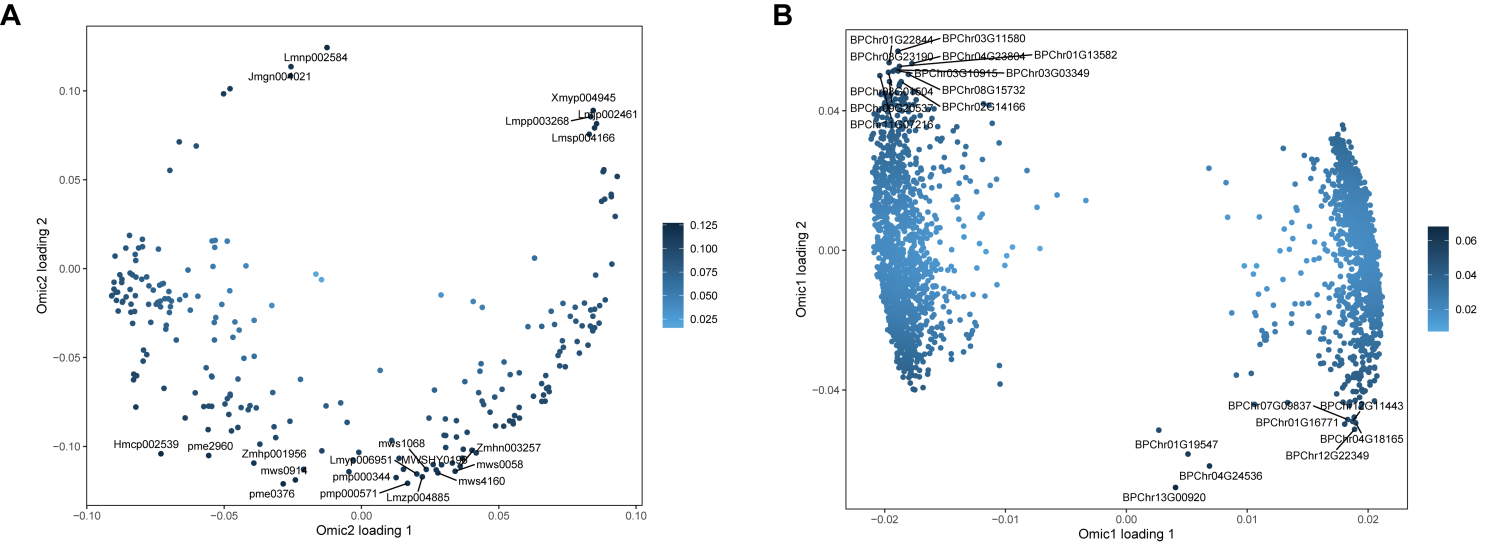


**Figure S6.** O2PLS analysis integrating flavonoid metabolites and MEblue module genes.


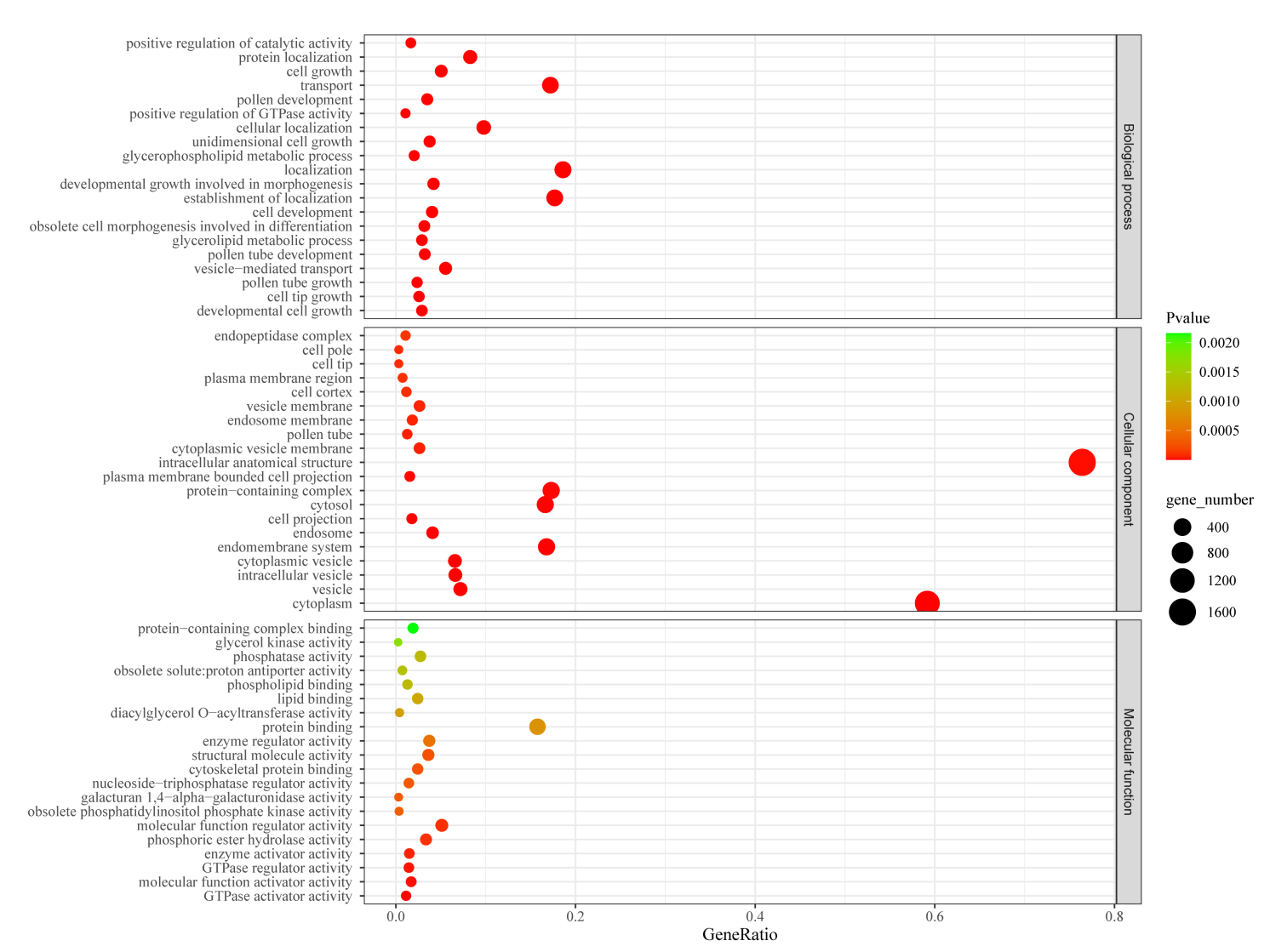


**Figure S7.** Gene Ontology (GO) analysis revealed the top 20 GO enrichments for the 'MEblue module' genes involved in biological processes, cellular components, and molecular functions between Coated and Control groups..

**
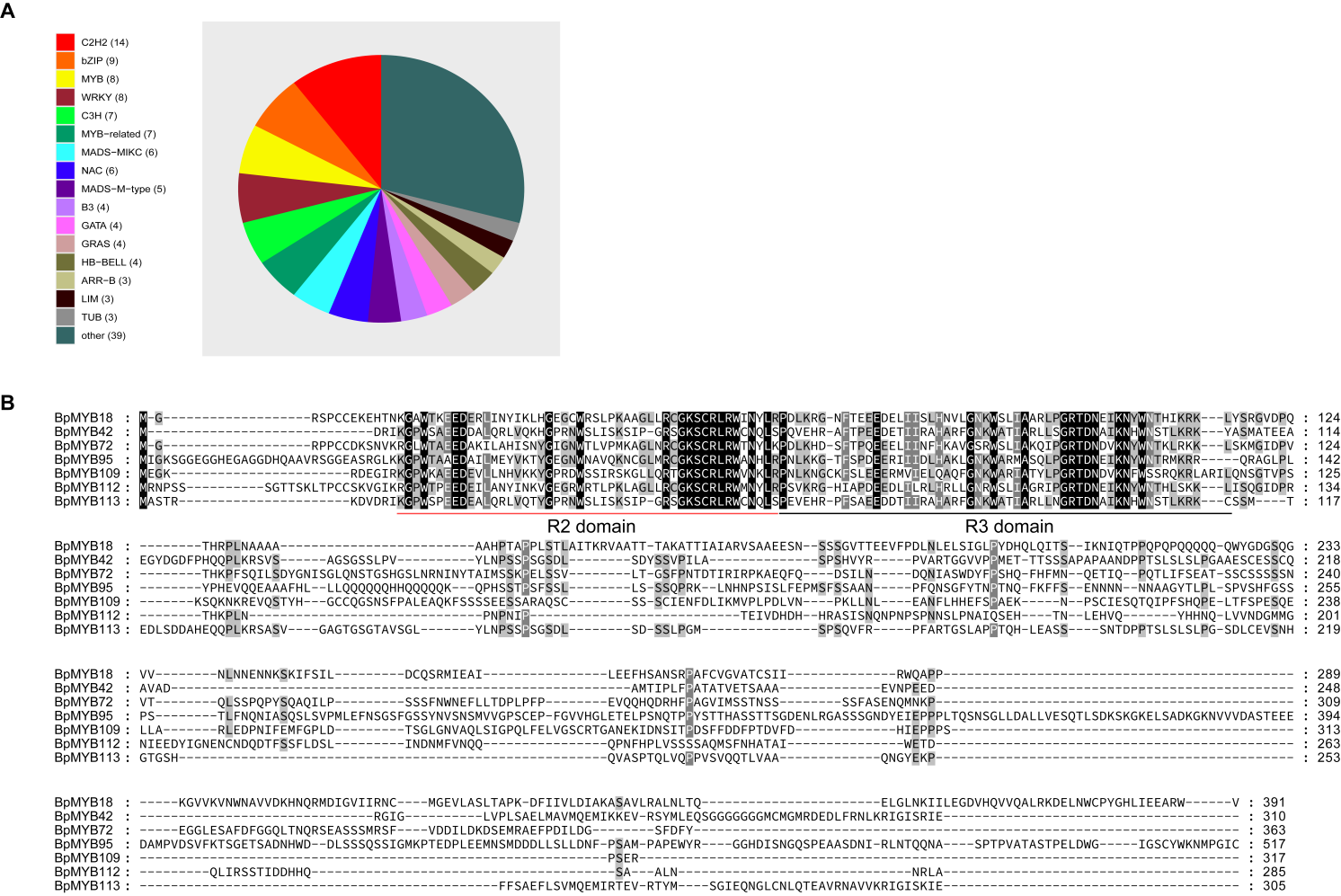
**

**Figure S8.** MEblue module TFs analysis. (A) Classification of MEblue module genes family; (B) Multiple amino-acid sequence alignment of candidate MYBs in MEblue module. protein sequence alignment. The alignment was generated using MAFFT v7.526, The R2 and R3 domain are marked below the alignment.


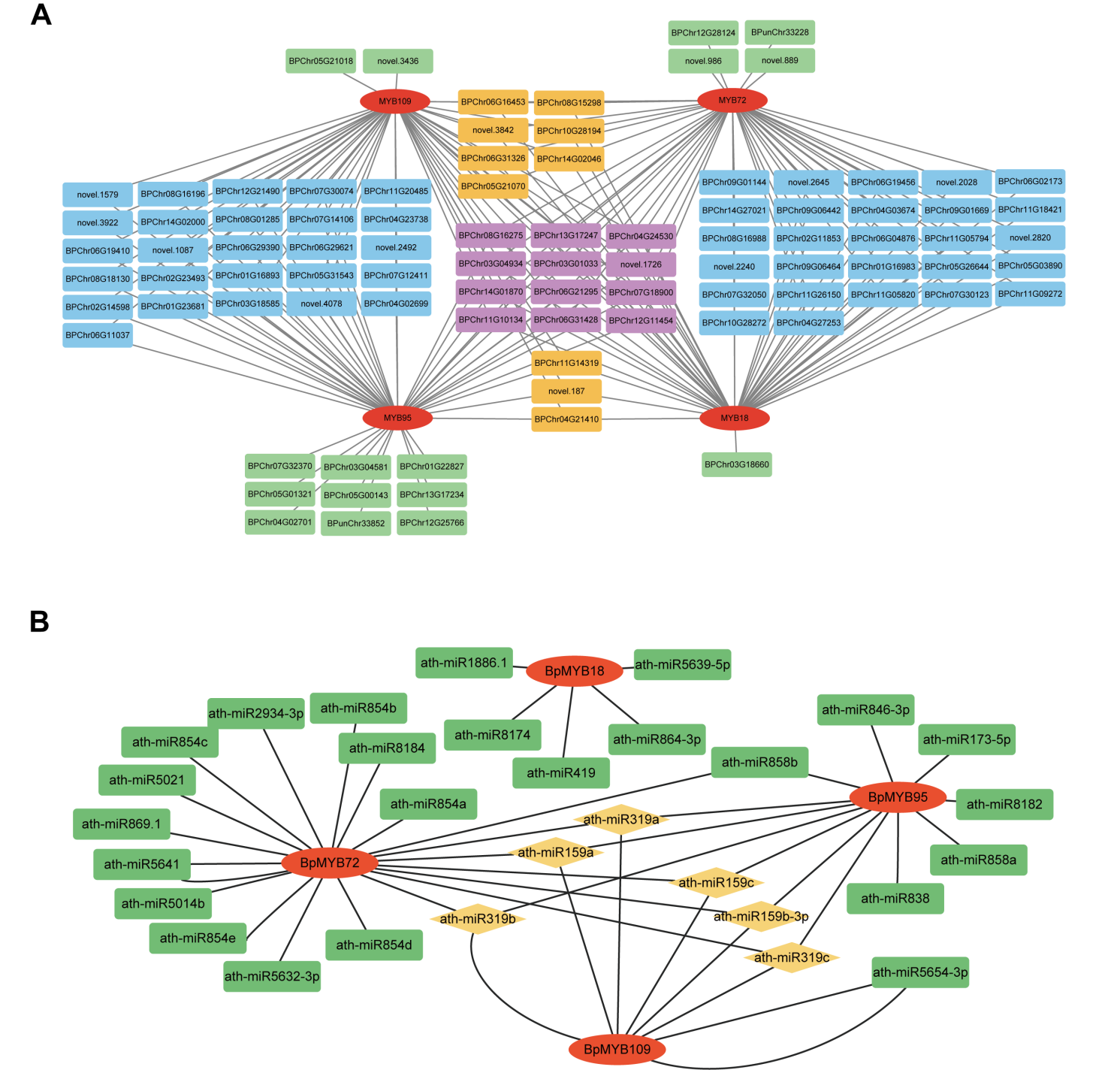


**Figure S9.** Co-expression network analysis. (A) Interaction analyses between *BpMYB18*, *BpMYB72*, *BpMYB95*, and Bp*MYB109*, and the MEblue module protein. Green, blue, yellow and purple boxes indicate shared interactions with one, two, three and four BpMYBs, respectively. BpMYBs highlighted in red box. (B) miRNA regulatory network of BpMYBs represented using Cytoscape software. Red box: BpMYBs; Yellow box: mRNAs co-expressed with three BpMYBs; Green box: miRNAs co-expressed with one BpMYB.

TABLE S1

Metabolite statistics list of differentially expressed in the four comparative combinations.

| Compounds | Class | Compound ID | Pathway ID |
| --- | --- | --- | --- |
| 2-α-Linolenoyl-glycerol-1,3-di-O-glucoside | Lipids | - | - |
| Myricetin-3-O-xyloside | Flavonoids | - | - |
| Epigallocatechin | Flavonoids | C12136 | ko00941, ko01110 |
| Epicatechin-4'-O-β-D-glucopyranoside | Flavonoids | - | - |
| Epicatechin-3'-O-β-D-glucopyranoside | Flavonoids | - | - |
| Vanillic Acid-4-O-Glucuronide | Phenolic acids | - | - |
| N1-Dihydrocaffeoyl-N10-coumaroylspermidine | Alkaloids | - | - |
| 2,2-Dimethylsuccinic acid | Organic acids | - | - |
| 5,7,4'-trihydroxy-8,3'-dimethoxyflavone | Flavonoids | - | - |
| Luteolin-7-O-neohesperidoside | Flavonoids | C12630 | ko00944 |
| 2-Hydroxyisobutyric acid | Organic acids | C21297 | - |
| Nootkatol | Terpenoids | - | - |
| 3,3',5,7-Tetrahydroxy-4',6-Dimethoxyflavone | Flavonoids | - | - |
| N-Hydroxytryptamine | Alkaloids | C17203 | ko00380, ko01100 |
| Gallocatechin | Flavonoids | C12127 | ko00941, ko01110 |

–: The metabolite was not annotated to a pathway.

TABLE S2

Flavonoid metabolite list of “MEblue” module under the WGCNA analysis.

| Compounds | Compound ID | Pathway ID |
| --- | --- | --- |
| Kaempferol-3-O-rutinoside | C21833 | ko00944, ko01110 |
| Luteolin-7-O-neohesperidoside | C12630 | ko00944 |
| 5,7,3',4'-Tetrahydroxyflavone | C01514 | ko0094, ko00944, ko01100, ko01110 |
| Hesperetin | C01709 | ko00941 |
| 3-O-Methylquercetin | C04443 | ko00944 |
| Quercetin-3-O-rutinoside | C05625 | ko00944, ko01100, ko01110 |
| Acacetin | C01470 | ko00944 |
| 3,7-O-Dimethylquercetin | C01265 | ko00944 |
| Apigenin-7-O-glucoside | C04608 | ko00944 |
| Quercetin-3-O-sophoroside | C12667 | ko00944 |

TABLE S3

A list of MYB-binding elements in the promoter of flavonoid biosynthesis pathway structural genes.

| ID | sequence | start position | length |
| --- | --- | --- | --- |
| BPChr06G11016(PAL) | CAACGG | 1723 | 6 |
| BPChr06G11016(PAL) | CAACTG | 1006 | 6 |
| BPChr06G11016(PAL) | CAACTG | 1234 | 6 |
| BPChr06G30228(PAL) | CAACTG | 1568 | 6 |
| BPChr06G31077(PAL) | CAACTG | 1568 | 6 |
| BPChr13G10287(CHS) | aaaAaaC(G/C)GTTA | 1734 | 10.5 |
| BPChr06G29586(CHS) | CAACTG | 188 | 6 |
| BPChr06G29586(CHS) | aaaAaaC(G/C)GTTA | 1321 | 10.5 |
| BPChr13G10350(FLS) | CAACGG | 1888 | 6 |
| BPChr13G10398(FLS) | CAACGG | 1888 | 6 |
| BPChr11G26387(CYP75A) | CAACTG | 1086 | 6 |
| BPChr11G26387(CYP75A) | CAACTG | 1618 | 6 |
| BPChr11G26387(CYP75A) | AACCTAA | 177 | 7 |
| BPChr04G05383(CYP75B1) | CAACTG | 238 | 6 |
| BPChr04G05383(CYP75B1) | AACCTAA | 1836 | 7 |

TABLE S4

Primer sequence of candidate MYB genes for qRT-PCR.

| Gene | Gene name | F' primers | R' primers |
| --- | --- | --- | --- |
| BPChr11G09309 | BpTUB | GACATCTGCTTCCGTACTCTGA | GGTACTGCTGTGATCCTCTTGA |
| BPChr03G09649 | BpMYB18 | GTGGAGTGACGACGGAGGAAGT | CTGCGGCGGCGTTTGTATGT |
| BPChr11G05659 | BpMYB72 | ACTGCCGAGGAGGACGCAAA | TGCTTGAGGTCAGGCTTGAGGT |
| BPChr11G28685 | BpMYB95 | ACGCCGCCATACTCGACAACT | CCACCAGGAGAGCATCCAGCAA |
| BPChr13G10264 | BpMYB109 | GAAAGTCGTGCCGCCTTCGTT | TCCCAAACTGTGCCTGCAACTC |
