# Supplementary material for: Integrated metabolome-transcriptome analyses reveal key pathways regulating staminate catkin development and pollen maturation in Betula platyphylla
Source: Front Plant Sci. 2025 May 21;16:1581560. doi: 10.3389/fpls.2025.1581560 (PMC12133839; doi:10.3389/fpls.2025.1581560)
Supplement: Supplementary file 2 [file Table1.docx]

TABLE S1

Metabolite statistics list of differentially expressed in the four comparative combinations.

| Compounds | Class | Compound ID | Pathway ID |
| --- | --- | --- | --- |
| 2-α-Linolenoyl-glycerol-1,3-di-O-glucoside | Lipids | - | - |
| Myricetin-3-O-xyloside | Flavonoids | - | - |
| Epigallocatechin | Flavonoids | C12136 | ko00941, ko01110 |
| Epicatechin-4'-O-β-D-glucopyranoside | Flavonoids | - | - |
| Epicatechin-3'-O-β-D-glucopyranoside | Flavonoids | - | - |
| Vanillic Acid-4-O-Glucuronide | Phenolic acids | - | - |
| N1-Dihydrocaffeoyl-N10-coumaroylspermidine | Alkaloids | - | - |
| 2,2-Dimethylsuccinic acid | Organic acids | - | - |
| 5,7,4'-trihydroxy-8,3'-dimethoxyflavone | Flavonoids | - | - |
| Luteolin-7-O-neohesperidoside | Flavonoids | C12630 | ko00944 |
| 2-Hydroxyisobutyric acid | Organic acids | C21297 | - |
| Nootkatol | Terpenoids | - | - |
| 3,3',5,7-Tetrahydroxy-4',6-Dimethoxyflavone | Flavonoids | - | - |
| N-Hydroxytryptamine | Alkaloids | C17203 | ko00380, ko01100 |
| Gallocatechin | Flavonoids | C12127 | ko00941, ko01110 |
| Kaempferol-3-O-(2''-O-acetyl)glucoside | Flavonoids | - | - |

–: The metabolite was not annotated to a pathway.
